# Care Practices, Morbidity and Mortality of Preterm Neonates in China, 2013–2014: a Retrospective study

**DOI:** 10.1038/s41598-019-56101-x

**Published:** 2019-12-27

**Authors:** Fengdan Xu, Xiangyong Kong, Shunyan Duan, Hongyan Lv, Rong Ju, Zhankui Li, Shujuan Zeng, Hui Wu, Xuefeng Zhang, Weipeng Liu, Fang Liu, Hongbin Cheng, Yanjie Ding, Tieqiang Chen, Ping Xu, Xiaomei Tong, Zhichun Feng

**Affiliations:** 1 Department of Neonatal Intensive Care Unit, Guangdong Medical University Affiliated Dongguan Children’s Hospital, Dongguan, 523325 China; 2 Department of Pediatrics, Guangdong Medical University, Dongguan, 523808 China; 3 Newborn Care Center, BaYi Children’s Hospital, The Seventh Medical Center of PLA General Hospital, Beijing, 100700 China; 4 Department of Neonatology, Guangdong Women and Children Hospital, Guangzhou, 510010 China; 5 Department of Neonatology, Handan Maternal and Child Health-Care Hospital, Handan, 056001 China; 6 Department of Neonatology, Chengdu Women’s and Children’s Central Hospital, Chengdu, 610091 China; 7 Department of Neonatology, North-West Women and Children’s Hospital, Xi’an, 710061 China; 8 Department of Neonatology, Longgang District Central Hospital of Shenzhen, Shenzhen, 518116 China; 9 Department of Neonatology, The First Hospital of Jilin University, Changchun, 130021 China; 10 Department of Neonatology, 302 Hospital of PLA, Beijing, 100039 China; 11 Department of Pediatrics, Navy General Hospital, Beijing, 100048 China; 12 Department of Neonatology, Bethune International Peace Hospital, Shijiazhuang, 050082 China; 13 Department of Pediatrics, Huangshi Women’s and Children’s Hospital, Huangshi, 435003 China; 14 Department of Pediatrics, Yantai Yuhuangding Hospital, Yantai, 264000 China; 15 Department of Neonatology, Changsha Hospital for Maternal and Child Health Care, Changsha, 410007 China; 16 Department of Neonatal Intensive Care Unit, Liaocheng People’s Hospital, Liaocheng, Shandong Prov 252000 China; 17 Department of Pediatrics, Peking University Third Hospital, Beijing, 100191 China

**Keywords:** Quality of life, Risk factors

## Abstract

This retrospective cohort study aimed to investigate the prevalence, morbidity, mortality and the maternal/neonatal care of preterm neonates and the perinatal risk factors for mortality. We included data on 13,701 preterm neonates born in 15 hospitals for the period 2013–2014 in China. Results showed a prevalence of preterm neonates of 9.9%. Most infants at 24–27 weeks who survived more than 12 hours were mechanically ventilated (56.1%). Few infants born before 28 weeks received CPAP without first receiving mechanical ventilation (8.1%). Few preterm neonates received antenatal steroid(35.8% at 24–27 weeks, 57.9% at 28–31 weeks, 57.0% at 32–33 weeks and 32.7% at 34–36 weeks). Overall mortality was 1.9%. Most of the deaths at 24–27 weeks of gestation occurred within 12 hours after birth, accounting for 68.1%(32/47), and within 12–72 hours after birth at 28–36 weeks of gestation, accounting for 47.4%(99/209). Rates of survival to discharge increased from 68.2% at 24–27 weeks, 93.3% at 28–31 weeks, 99.2% at 32–33 weeks to 99.4% at 34–36 weeks. The smaller of the GA, there was a greater risk of morbidities due to prematurity. Preterm birth weight (*OR* = 0.407, 95% *CI* 0.346–0.478), antenatal steroid (*OR* = 0.680, 95% *CI* 0.493–0.938), and neonatal asphyxia (*OR* = 3.215, 95% *CI* 2.180–4.741) proved to significantly influence the odds of preterm neonatal death. Overall, our results support that most of the preterm neonates at 28–36 weeks of gestation survived without major morbidity. Rate of survival of GAs less than 28 weeks was still low. Maternal and infant care practices need to be improved in the very preterm births.

## Introduction

Premature birth was the main cause of death in the first month of life and it also was a factor in greater than 75% of early deaths in the neonatal period. It has been reported that the improvements in outcomes of preterm neonates has increased, with significant differences between centers around the world^1–8^. However, preterm neonates continue to contribute a leading cause of mortality, morbidity, and long-term neurodevelopmental disability. It is important to evaluate the current morbidity and mortality data of the premature infants for counseling families and considering novel interventions to improve outcome. This study aimed to analyze outcomes in antenatal and postnatal care, morbidities, mortality and the perinatal risk factors for preterm neonatal mortality among preterm neonates born in 15 hospitals in China from January 1, 2013 to December 31, 2014.

## Methods

We established a collaborative study group including nine general hospitals and six maternal and children’s hospitals from major cities of 11 provinces in China^5^. All the participating hospitals were tertiary neonatal care centers and had their own maternity local wards that represent academic institutions with large obstetric and neonatal services, expertise in caring for high-risk mothers and preterm infants, and experience in multicenter clinical research. In this retrospective cohort study design, data collection for this study was approved by the Institutional Ethics Committee of the Army General Hospital of the People’s Liberation Army (PLA) (number 2012–12) and adopted by each center according to Chinese regulations. The study was also conducted as part of the Neonatal Research Network of the Chinese Neonatologist, which is a sub-association of the Chinese Medical Doctor Association. Coordination for this study was based at the Army General Hospital of PLA.

Data were collected prospectively with maternal pregnancy and delivery information collected soon after birth, and infant data collected until death, hospital discharge/transfer. Infants who died in the first 12 hours of life were included in analyses of overall mortality but were excluded from analyses focuses on morbidities and the use of pulmonary surfactant and respiratory support as these outcomes were limited for infants who died within 12 hours. Morbidities diagnosed during the initial hospital stay were recorded for infants who survived more than 12 hours.

We focused on maternal and neonatal care practices and neonatal morbidity and mortality^9^. Changes in maternal/neonatal characteristics, including maternal age, antenatal fever, rupture of membrane, chorioamnionitis, placental abruption, placenta previa, pregnancy-induced hypertension, gestational diabetes mellitus, infant gestational age (GA), birth weight (BW), multiple birth and neonatal asphyxia that might influence outcomes were examined. Care practices reported were chosen because they have been associated with neonatal outcomes and included antenatal steroids, cesarean delivery, surfactant therapy, and respiratory support. Morbidities included necrotizing enterocolitis (NEC), stage 1–3^10,11^; early (≤72 hours) and late-onset (>72 hours) sepsis, defined by cultures positive for bacteria or fungi, and antibiotic therapy ≥5 days or intent to treat but death <5 days^12,13^; intracranial hemorrhage (ICH); intraventricular hemorrhage(IVH); cystic periventricular leukomalacia (PVL); retinopathy of prematurity (ROP) among infants hospitalized at 28 days; and bronchopulmonary dysplasia (BPD), defined as oxygen use at 36 weeks postmenstrual age or at discharge/transfer if before 36 weeks in infants who survived to 36 weeks. ICH was based on the most severe cranial sonogram prior to hospital discharge, transfer, or death. Grade 3/4 IVH was considered severe^14^. Survival to discharge and survival without major morbidity (moderate/severe BPD, IVH stage ≥III, cystic PVL, NEC stage III, ROP grade ≥ 3, and early-onset and late-onset sepsis) were studied. Neonatal asphyxia is the inability of a newborn to initiate and sustain respiration. Apgar score should be combined with blood gas results to make the diagnosis of neonatal asphyxia: (1) Mild asphyxia: Apgar score 1 min ≤ 7, or 5 min ≤ 7, with umbilical artery pH < 7.2; (2) severe asphyxia: Apgar Score 1 min ≤ 3 or 5 min ≤ 5, with umbilical artery blood PH < 7.0.

### Statistical analysis

The EPIDATA database was used for datasheet recordings, and statistical analysis was performed using SPSS software (v. 19.0). Descriptive statistical methods were used to describe the study population. Numerical data are expressed as the mean ± S.D. Comparison between continuous variables was conducted by using a One-way ANOVA. Categorical variables are presented as frequencies or rates and were compared using Chi-square test or Fisher’s exact tests wherever appropriate. Two binary logistic regression models (infant and maternal variables) were constructed to identify predictors of preterm neonatal mortality. After adjusting for GA and 5 minute’s Apgar score, BW, gender, maternal perinatal complications such as: delivery mode, multiple birth, antenatal steroid, antenatal fever before delivery, rupture of membrane, chorioamnionitis, pregnancy-induced hypertension, gestational diabetes mellitus and neonatal asphyxia were included in the analysis. A 2-sided *P*-value < 0.05 was considered significant.

## Results

### Prevalence of preterm neonates

**Figure 1:**
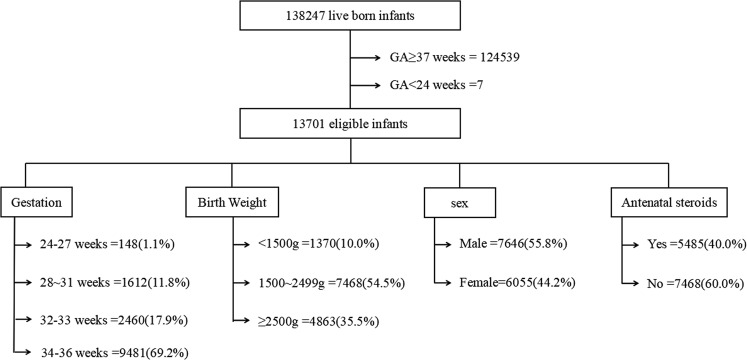
Flow chart of the study population. During 2013–2014, 138,247 alive neonates, which including 13,701 preterm neonates were born at 15 participating hospitals.

During 2013–2014, 138,247 alive neonates, which including 13,701 preterm neonates were born at 15 participating hospitals. The prevalence of preterm neonates was 9.9%. Neonates’ mean gestational age was 34.4 weeks (maximum:36; minimum:24). The gestational age was ≤31 weeks in 12.8%(n: 1,760) and ≤27 weeks in 1.1%(n: 148). Neonates’ mean birth weight was 2250.7 grams (maximum:4225; minimum:400). Birth weight was less than 2500 grams in 64.5% (n:8838) and less than 1500 grams in 10.0% (n:1370). See Fig. 1.

### Maternal/neonatal characteristics and care practices

Overall, there were 7,646(55.8%) male neonates, 4535(33.1%) multiple births, and 1650(12.6%) suffered from neonatal asphyxia. Caesarean sections were done in 8198(59.8%) neonates and the rate was increased with increased GA, with 16.2% at 24–27 weeks and 64.1% at 34–36 weeks. Rate of pulmonary surfactant use increased with decreased GA, from 57.4% at 24–27 weeks to 3.2% at 34–36 weeks. Most infants at 24–27 weeks who survived more than 12 hours were mechanically ventilated(56.1%). Few infants born before 28 weeks received CPAP without first receiving mechanical ventilation (8.1%). Few infants born at 24–27 weeks received antenatal steroid(35.8%). Antenatal steroid was given most for preterm labor between 28–31(57.9%) weeks and 32–33(57.0%) weeks of gestation.

Spontaneous labor without rupture of membranes accounts for more than half of the cases of preterm labor(66.1%). Some preterm deliveries are medically indicated due to health risks to mother and fetus including: chorioamnionitis, placental abruption, placenta previa, pregnancy-induced hypertension, gestational diabetes mellitus or abnormality of the amniotic fluid, see Tables 1–3.

**Table 1:** Maternal characteristics of the study population.

Characteristic	24–27 weeks (n = 148)	28–31 weeks (n = 1612)	32–33 weeks (n = 2460)	34–36 weeks (n = 9481)	Total (N = 13701)	*F*/*χ*^*2*^	*p*
Age, mean (SD),y	28.8 (5.2)	29.1 (5.3)	29.0 (5.1)	29.0 (4.8)	29.0 (4.9)	0.329	0.804
Antenatal steroid[n (%)]	53 (35.8)	933 (57.9)	1402 (57.0)	3097 (32.7)	5485 (40.0)	724.027	0.000
Antenatal fever[n (%)]	39 (26.4)	318 (20.4)	502 (21.2)	1746 (19.4)	2605 (19.9)	8.222	0.042
Rupture of membrane[n (%)]	46 (31.1)	628 (40.2)	1019 (43.0)	3135 (34.8)	4828 (36.9)	65.414	0.000
Chorioamnionitis[n (%)]	6 (4.1)	38 (2.5)	44 (1.9)	128 (1.5)	216 (1.7)	14.158	0.003
Placental abruption[n (%)]	9 (6.1)	89 (5.7)	120 (5.1)	181 (2.0)	399 (3.0)	107.513	0.000
Placenta previa[n (%)]	7 (4.7)	95 (6.1)	106 (4.5)	472 (5.2)	680 (5.2)	5.073	0.167
Pregnancy-induced hypertension[n (%)]	5 (3.4)	171 (10.9)	334 (14.1)	943 (10.5)	1453 (11.1)	34.438	0.000
Gestational diabetes mellitus[n (%)]	3 (2.0)	111 (7.1)	187 (7.9)	847 (9.4)	1148 (8.8)	20.444	0.000
Abnormality of the amniotic fluid[n (%)]	11 (9.4)	141 (11.4)	201 (10.9)	589 (8.2)	942 (9.1)	22.151	0.000

**Table 2:** neonatal characteristics of the study population.

Characteristic	24–27 weeks (n = 148)	28–31 weeks (n = 1612)	32–33 weeks (n = 2460)	34–36 weeks (n = 9481)	Total (N = 13701)	F/χ^2^	p
Male[n (%)]	88 (59.5)	913 (56.6)	1398 (56.8)	5247 (55.3)	7646 (55.8)	3.124	0.373
GA*,mean (SD), wk	26.6 (1.1)	30.4 (1.1)	33.0 (0.6)	35.6 (0.9)	34.4 (2.2)	24625.8	0.000
BW^#^, mean (SD), g	949.9 (287.1)	1470.8 (334.9)	1930.5 (373.7)	2486.7 ()458.7	2250.7 (575.7)	3631.2	0.000
Cesarean delivery[n (%)]	24 (16.2)	683 (42.4)	1412 (57.4)	6079 (64.1)	8198 (59.8)	400.206	0.000
Multiple birth[n (%)]	56 (37.8)	458 (28.4)	767 (31.2)	3254 (34.3)	4535 (33.1)	27.986	0.000
Neonatal asphyxia[n (%)]	93 (62.8)	543 (34.8)	408 (17.2)	606 (6.7)	1650 (12.6)	1365.210	0.000

*GA: gestational age, ^#^BW:birth weight.

**Table 3:** Care practices of the study population.

Characteristic	24–27 weeks n = 148)	28–31 weeks (n = 1612)	32–33 weeks (n = 2460)	34–36 weeks (n = 9481)	Total (N = 13701)	F/χ^2^	p
Pulmonary surfactant	85 (57.4)	658 (40.8)	443 (18.0)	307 (3.2)	1493 (10.9)	2517.415	0.000
Any high-frequency ventilation or conventional ventilation	83 (56.1)	574 (35.6)	379 (15.4)	365 (3.8)	1401 (10.2)	1962.105	0.000
Any nasal SIMV or CPAP therapy	53 (35.8)	780 (48.4)	779 (31.7)	871 (9.2)	2483 (18.1)	1840.568	0.000
Nasal SIMV or CPAP therapy highest*	12 (8.1)	392 (24.3)	521 (21.2)	650 (6.9)	1575 (11.5)	689.484	0.000

*Highest level of support was defined for nasal SIMV as never used conventional for high-frequency ventilation but used nasal SIMV, and for CPAP as never used conventional or high-frequency ventilation or nasal SIMV but received CPAP therapy.

### Morbidity and Survival

**Figure 2:**
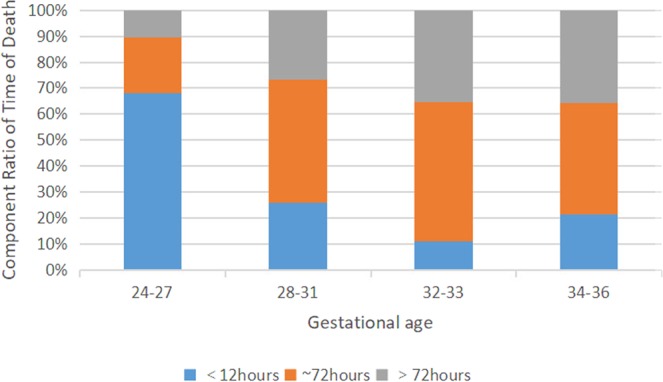
Distribution of time of death in premature infants of different gestational ages.

Deliveries occurring prior to 37 weeks place neonates at increased risks for mortality and morbidity. According to the information provided by our collaborative study group, overall mortality among preterm neonates was 1.9%(256/13701). Most of the deaths at 24–27 weeks of gestation occurred within 12 hours after birth, accounting for 68.1%(32/47), and within 12–72 hours after birth at 28–36 weeks of gestation, accounting for 47.4%(99/209). See Fig. 2. The smaller of the GA, the higher the mortality was. In total, there was no neonate survived at 24 weeks gestation. Rates of survival to discharge increased with the increase of GA, from 68.2% at 24–27 weeks, to 99.4% at 34–36 weeks. See Table 4.

**Table 4:** Survival to discharge for infants born at GA 24–36 weeks in 15 hospitals in China January 1, 2013-December 31, 2014.

No./Total(%)	24–27 weeks (n = 148)	28–31 weeks (n = 1612)	32–33 weeks (n = 2460)	34–36 weeks (n = 9481)	Total (N = 13701)	χ^2^	p
Died	47 (31.8)	108 (6.7)	45 (1.8)	56 (0.6)	256 (1.9)	1010.711	0.000
Survived to discharge^a^	101 (68.2)	1504 (93.3)	2415 (99.2)	9425 (99.4)	13445 (98.1)	1010.711	0.000
Survived to discharge without major morbidity^b^	64 (63.4)	1246 (82.8)	2227 (92.2)	9137 (96.9)	12674 (94.3)	685.124	0.000
RDS^a^	131 (88.5)	900 (55.8)	603 (24.5)	512 (5.4)	2146 (15.7)	3465.331	0.000
RDS stage III+^a^	50 (33.8)	178 (11.0)	115 (4.7)	81 (0.9)	424 (3.1)	3688.745	0.000
BPD^c^	29 (19.6)	165 (10.2)	37 (1.5)	54 (0.6)	285 (2.1)	859.508	0.000
Moderate/severe BPD^c^	15 (10.1)	83 (5.1)	21 (0.8)	48 (0.5)	167 (1.3)	898.967	0.000
ICH^c^	33 (22.3)	227 (14.1)	293 (11.9)	639 (6.7)	1192 (8.7)	171.016	0.000
Severe IVH^c^	6 (4.1)	10 (0.6)	7 (0.3)	8 (0.1)	31 (0.2)	260.424	0.000
cystic PVL^c^	1^e^	13 (0.8)	1^e^	3^e^	18 (0.1)	—^d^	0.000
NEC^c^	8 (5.4)	57 (3.5)	45 (1.8)	60 (0.6)	170 (1.2)	125.795	0.000
NEC stage III^c^	1^e^	5^e^	1^e^	1^e^	8^e^	—^d^	0.169
ROP^c^	17 (11.5)	69 (4.3)	20 (0.8)	38 (0.4)	144 (1.1)	356.511	0.000
ROP stage III+^c^	3^e^	9^e^	2^e^	7^e^	21 (1.5)	—^d^	0.790
Sepsis^a^	20 (13.5)	147 (9.1)	114 (4.6)	131 (1.4)	412 (3.0)	370.685	0.000

RDS respiratory distress syndrome, including grade I, II, III and IV, BPD bronchopulmonary dysplasia, including mild, moderate and severe BPD, ICH intracranial hemorrhage, IVH intraventricular hemorrhage, Severe IVH including grade III and IV, NEC necrotizing enterocolitis, including stage I, II and III, ROP retinopathy of prematurity, including grade 1, 2, 3, 4 and 5.

^a^Proportion among all infants including survivors.

^b^Major morbidity was defined as one or more of moderate/severe BPD, IVH stage ≥ III, cystic PVL, NEC stage III, ROP grade ≥ 3, and sepsis (early-onset sepsis, late-onset sepsis).

^c^Proportion among infants who survived >7 days.

^d^Fisher’s exact tests.

^e^Numerator less than 10.

The smaller of the GA, there was a greater risk of morbidity due to prematurity, including the morbidity of RDS and severe RDS, BPD and moderate/severe BPD, ICH and severe IVH, NEC, ROP and sepsis. There was a significant increase in survival without major neonatal morbidity for neonates born at 28 to 36 weeks. But only 63.4% neonates less than 28 weeks GA survived without major neonatal morbidity. See Table 4.

### Perinatal risk factors for preterm neonatal mortality

Table 5 summarizes all significant perinatal risk factors for preterm neonatal mortality, as revealed by multivariable logistic regression modeling. Preterm birth with lower birth weight showed a major impact on the mortality after adjusting for gestational age and 5 minute’s Apgar score (*OR* = 0.407, 95% *CI* 0.346–0.478). Women not having received antenatal steroid were at higher odds of neonatal death, compared to women covered by prevention efforts (*OR* = 0.680, 95% *CI* 0.493–0.938). Neonatal asphyxia was significantly associated with neonatal mortality (*OR* = 3.215, 95% *CI* 2.180–4.741). However, gender, caesarean section, multiple birth, antenatal fever before delivery, rupture of membrane, chorioamnionitis, pregnancy-induced hypertension and gestational diabetes mellitus proved by our study to be insignificant variables.

**Table 5:** Multivariate logistic regression analysis of risk factors for mortality.

Factors	B value	Wald	OR^#^	95%CI	P
BW	−0.899	118.948	0.407	0.346–0.478	0.000
Male	0.178	1.253	1.195	0.875–1.632	0.263
Caesarean section	0.237	1.697	1.268	0.887–1.813	0.193
Multiple birth	0.040	0.051	1.041	0.737–1.469	0.821
Neonatal asphyxia	1.168	34.736	3.215	2.180–4.741	0.000
Antenatal steroid	−0.386	5.539	0.680	0.493–0.938	0.019
Antenatal fever before delivery	0.188	1.054	1.207	0.843–1.730	0.305
Rupture of membrane	−0.195	1.192	0.823	0.580–1.167	0.275
Chorioamnionitis	−0.414	0.499	0.661	0.210–2.085	0.480
Pregnancy-induced hypertension	0.128	0.328	1.136	0.734–1.758	0.567
Gestational diabetes mellitus	−0.329	0.691	0.720	0.331–1.563	0.406

GA: gestational age, BW: birth weight.

^#^After adjusting for gestational age and 5 minute’s Apgar score.

## Discussion

Preterm birth is one of the most challenging problems in obstetric care and it is closely related to perinatal mortality and morbidity. Although in the past few decades, neonatal intensive care has made continuous progress and neonatal mortality has declined significantly, the morbidity and mortality among preterm neonates still have a lot of room for improvement. The objective of the present study was to investigate the prevalence of preterm neonates and the maternal/neonatal care, complications, mortality and the risk factors for preterm neonatal mortality in China. The results show that progress is being made and outcomes of the preterm neonates are improving. But rate of survival of GAs less than 28 weeks was still low. Maternal and infant care practices need to be improved in the very preterm births.

The survival rates of preterm neonates with GA of 25, 26 and 27 weeks were 75.6%, 85.1% and 91.1%, respectively, in Australia in 2007–2011^2^. Similar rates of 72%, 84% and 88%, respectively, were observed in America in 2003–2007^7^. These rates were 0, 0 and 58.3%, respectively, in 2010^8^ and 41.7%, 91.3% and 92.5%, respectively, in 2012 in China^15^. The current survey showed a significant increase in survival to discharge for preterm neonates, especially for those born at 25 to 27 weeks, compared with previous Chinese reports. But this rate was still low compared with the developed countries. Good obstetric and neonatal care is an important factor in reducing morbidities and improving neonatal outcomes. Our study fined a significant increase in survival without major neonatal morbidity for neonates born at 28 to 36 weeks. Although overall survival increased for neonates 25 to 27 weeks, only 63.4% neonates less than 28 weeks GA survived without major neonatal morbidity, highlighting the continued need for interventions to improve outcomes for the most immature neonates.

Previous studies have shown that antenatal steroids use can improve the perinatal health in preterm neonates^16–21^. The 1995 National Institutes of Health consensus statement on antenatal corticosteroids led to widespread use. Recent studies have documented benefits to neonates with GA less than 34 weeks. This study showed that women not having received antenatal steroid were at higher odds of neonatal death, compared to women covered by prevention efforts (*OR* = 0.680, 95% *CI* 0.493–0.938). In China, dexamethasone was the only steroid used, and it is recommended that a course of antenatal corticosteroids should be given to mothers before delivery between 24 weeks to 34 weeks gestation. But our study shows that antenatal corticosteroids uptake is still very low, similar to other low-income and middle-income countries^22^. Suggesting that there is an urgent need to increase the use of antenatal corticosteroids before preterm deliveries in China in order to improve preterm survival and morbidity.

Less aggressive ventilation and other strategies that can reduce lung injury are increasingly embraced. If early CPAP is sufficient to prevent alveolar collapse in some preterm neonates, intubation will be avoided. Our study showed that rate of pulmonary surfactant use increased with decreased GA, from 57.4% at 24–27 weeks to 3.2% at 34–36 weeks, and few infants(8.1%) born before 28 weeks received CPAP without first receiving mechanical ventilation.

The logistic regression model for the neonatal variables comprised of BW, gender, caesarean section, multiple birth, neonatal asphyxia, antenatal steroid, antenatal fever before delivery, rupture of membrane, chorioamnionitis, pregnancy-induced hypertension and gestational diabetes mellitus. After adjusting for GA and 5 minute’s Apgar score, this study demonstrated that the neonatal mortality is inversely proportional to birth weight. Antenatal corticosteroids are established as an effective method of reducing preterm morbidity and mortality. Neonatal asphyxia was significantly associated with neonatal mortality (*OR* = 3.215, 95% *CI* 2.180–4.741). Premature rupture of membrane(PROM) is the rupture of the amniotic membrane before the onset of labor^23^. In preterm pregnancy, premature rupture of membranes occurs in 2.0% to 3.5% of pregnancies. It is the most common cause of premature birth, which occurs in 30–40% of cases^24^. Most studies have found that PROM is associated with high maternal and perinatal morbidity and mortality risks. But in contrast, Pramod Pharande’s study show that late premature PROM at or after 24 weeks had lower CLD/mortality compared with No-PPROM^25^. Our study observed that gender, caesarean section, multiple birth, antenatal fever before delivery, rupture of membrane, chorioamnionitis, pregnancy-induced hypertension and gestational diabetes mellitus proved by our study to be insignificant variables.

Although similar studies may have been conducted elsewhere, differences in medical policies, disease types and clinical practices in different hospitals and countries will make the conclusions of these studies irrelevant to China experience. The results of this multicenter study will provide information on premature mortality and will help to reduce neonatal mortality and morbidity.

Limitations of the study entailed its short time period of two year for the study, and our cohort is hospital-based rather than population-based. Although the number is large, our cohort does not represent the whole premature population in China, but the premature population selected from the academic centers. A valuable extension to this study is to observe the long-term prognosis of the preterm neonates. But the follow-up data are not included in this study. Nevertheless, this is the first step in the interpretation and statistical analysis of data related to China. This would give us a more comprehensive understanding of how to conduct future research and can help to better allocate resources and institute preventative measures.

## Conclusions

Although mortality in preterm neonates, especially extremely preterm neonates decreased markedly in China, this group still contributes the major cause of neonatal deaths. Most of the preterm neonates at 28–36 weeks of gestation survived without major morbidity. Rate of survival of GAs less than 28 weeks was still low. Maternal and infant care practices need to be improved in the very preterm births.
